# 1-(Adamantan-1-yl)-3-(4-fluoro­phen­yl)thio­urea

**DOI:** 10.1107/S1600536812017515

**Published:** 2012-04-25

**Authors:** Güneş Demirtaş, Necmi Dege, Mona M. Al-Shehri, Ali A. El-Emam, Nasser R. El-Brollosy, Orhan Büyükgüngör

**Affiliations:** a Ondokuz Mayıs University, Arts and Sciences Faculty, Department of Physics, 55139 Samsun, Turkey; b Department of Pharmaceutical Chemistry, College of Pharmacy, King Saud University, 11451 Riyadh, Saudi Arabia

## Abstract

In the title mol­ecule, C_17_H_21_FN_2_S, the mean planes of the benzene ring and the thio­urea fragment form a dihedral angle of 61.93 (9)°. In the crystal, pairs of weak N—H⋯S inter­actions link the mol­ecules, forming inversion dimers.

## Related literature

For background to the biological activity of adamantane and thio­urea derivatives, see: Vernier *et al.* (1969 ▶); El-Emam *et al.* (2004 ▶); Li *et al.* (2009 ▶); Hunter *et al.* (2008 ▶); Kadi *et al.* (2007 ▶, 2010 ▶). For the crystal structures of related adamantane deriv­atives, see: Kadi *et al.* (2011 ▶); Almutairi *et al.* (2012 ▶); Al-Abdullah *et al.* (2012 ▶).
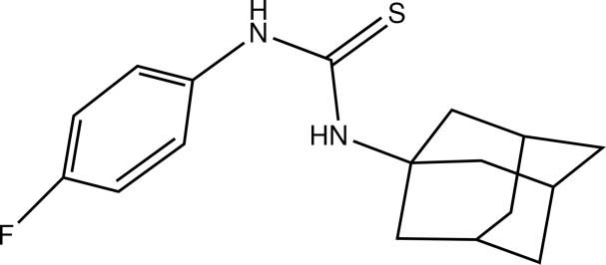


## Experimental

### 

#### Crystal data

C_17_H_21_FN_2_S
*M* *_r_* = 304.42
Triclinic, 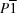

*a* = 6.4274 (5) Å
*b* = 11.4727 (9) Å
*c* = 11.5870 (9) Å
α = 113.510 (6)°
β = 94.721 (6)°
γ = 94.837 (6)°
*V* = 774.39 (10) Å^3^
*Z* = 2
Mo *K*α radiation
μ = 0.22 mm^−1^
*T* = 296 K
0.80 × 0.35 × 0.11 mm

#### Data collection

Stoe IPDS 2 diffractometer
Absorption correction: integration (*X-RED32*; Stoe & Cie, 2002 ▶) *T*_min_ = 0.847, *T*_max_ = 0.977
11154 measured reflections
3048 independent reflections
2224 reflections with *I* > 2σ(*I*)
*R*_int_ = 0.078

#### Refinement

*R*[*F* ^2^ > 2σ(*F* ^2^)] = 0.048
*wR*(*F* ^2^) = 0.118
*S* = 1.01
3048 reflections
190 parameters
H-atom parameters constrained
Δρ_max_ = 0.22 e Å^−3^
Δρ_min_ = −0.21 e Å^−3^

### 

Data collection: *X-AREA* (Stoe & Cie, 2002 ▶); cell refinement: *X-AREA*; data reduction: *X-RED32* (Stoe & Cie, 2002 ▶); program(s) used to solve structure: *SHELXS97* (Sheldrick, 2008 ▶); program(s) used to refine structure: *SHELXL97* (Sheldrick, 2008 ▶); molecular graphics: *ORTEP-3 for Windows* (Farrugia, 1997 ▶); software used to prepare material for publication: *WinGX* (Farrugia, 1999 ▶) and *PLATON* (Spek, 2009 ▶).

## Supplementary Material

Crystal structure: contains datablock(s) I, global. DOI: 10.1107/S1600536812017515/cv5285sup1.cif

Structure factors: contains datablock(s) I. DOI: 10.1107/S1600536812017515/cv5285Isup2.hkl

Supplementary material file. DOI: 10.1107/S1600536812017515/cv5285Isup3.cml

Additional supplementary materials:  crystallographic information; 3D view; checkCIF report

## Figures and Tables

**Table 1:** Hydrogen-bond geometry (Å, °)

*D*—H⋯*A*	*D*—H	H⋯*A*	*D*⋯*A*	*D*—H⋯*A*
N1—H1⋯S1^i^	0.86	2.67	3.3939 (19)	142

Symmetry code: (i) 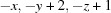.

**Fig. 1.**
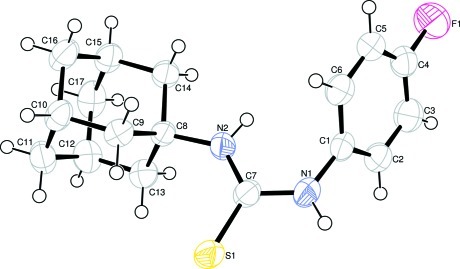
The molecular structure of (I) showing the atomic numbering scheme. Displacement ellipsoids are drawn at the 50% probability level.

## supplementary crystallographic information

### Comment

Derivatives of adamantane have long been known for their diverse biological activities including antiviral activity against the influenza (Vernier *et al.*, 1969) and HIV viruses (El-Emam *et al.*, 2004). Moreover, adamantane derivatives were recently reported to exhibit marked antibacterial and anti-inflammatory activities (Kadi *et al.*, 2007, 2010). In addition, 1,3-disubstituted thiourea derivatives were reported to possess potent antiviral activity (Li *et al.*, 2009; Hunter *et al.*, 2008). In continuation of our structural studies of adamantane derivatives (Kadi *et al.*, 2011; Al-Abdullah *et al.*, 2012; Almutairi *et al.*, 2012), we present here the crystal structure of the title compound, (I).

In (I) (Fig. 1), the adamantyl and fluorobenzene fragments are bridged by thiourea fragment. The C—C single bond distances range from 1.514 (4) Å to 1.537 (3) Å and C≐C bond distances for benzene ring range from 1.359 (4) Å to 1.383 (3) Å. The torsion angle of C6—C1—N1—C7 is 62.7 (3)° and the bond distance between the F and C atoms is 1.362 (3) Å. The weak intermolecular N1—H1···S1 hydrogen bond (Table 1) link two molecules into centrosymmetric dimer.

### Experimental

A mixture of 1-adamantylamine (1.51 g m, 0.01 mol) and 4-fluorophenyl isothiocyanate (1.53 g m, 0.01 mol), in ethanol (10 ml), was heated under reflux for 4 h. On cooling, the precipitated crude product was filtered, dried and crystallized from ethanol to yield 2.68 g m (88%) of the title compound (C_17_H_21_FN_2_S) as colourless crystals. M.P.: 169–171 *^o^*C. Single crystals suitable for X-ray diffraction were obtained by slow evaporation of a solution of the title compound in EtOH/CHCl_3_ (1:1) at room temperature. ^1^H NMR (DMSO-d_6_, 500.13 MHz): d 1.64 (s, 6H, Adamantane-H), 2.06 (s, 3H, Adamantane-H), 2.23 (s, 6H, Adamantane-H), 7.10–7.14 (m, 2H, Ar—H), 7.24 (s, 1H, NH), 7.43 (d, 2H, Ar—H, *J* = 6.5 Hz), 9.25 (s, 1H, NH). ^13^C NMR (DMSO-d_6_, 125.76 MHz): 28.98, 35.94, 40.80, 53.24 (Adamantane-C), 114.72, 125.49, 135.79, 157.66 (Ar—C), 179.06 (C=S).

### Refinement

All H atoms were positioned geometrically [N—H=0.860 Å and C—H=0.930 Å, 0.970 Å or 0.980 Å] and treated as riding with *U*_iso_(H)=1.2*U*_eq_(C,*N*).

### Crystal data

**Table:**

C_17_H_21_FN_2_S	*Z* = 2
*M**_r_* = 304.42	*F*(000) = 324
Triclinic, *P*1	*D*_x_ = 1.306 Mg m^−^^3^
Hall symbol: -P 1	Mo *K*α radiation, λ = 0.71073 Å
*a* = 6.4274 (5) Å	Cell parameters from 14319 reflections
*b* = 11.4727 (9) Å	θ = 3.2–27.9°
*c* = 11.5870 (9) Å	µ = 0.22 mm^−^^1^
α = 113.510 (6)°	*T* = 296 K
β = 94.721 (6)°	Prism, colourless
γ = 94.837 (6)°	0.80 × 0.35 × 0.11 mm
*V* = 774.39 (10) Å^3^	

### Data collection

**Table:**

Stoe IPDS 2 diffractometer	3048 independent reflections
Radiation source: fine-focus sealed tube	2224 reflections with *I* > 2σ(*I*)
Graphite monochromator	*R*_int_ = 0.078
rotation method scans	θ_max_ = 26.0°, θ_min_ = 3.2°
Absorption correction: integration (*X-RED32*; Stoe & Cie, 2002)	*h* = −7→7
*T*_min_ = 0.847, *T*_max_ = 0.977	*k* = −14→14
11154 measured reflections	*l* = −14→14

### Refinement

**Table:**

Refinement on *F*^2^	Primary atom site location: structure-invariant direct methods
Least-squares matrix: full	Secondary atom site location: difference Fourier map
*R*[*F*^2^ > 2σ(*F*^2^)] = 0.048	Hydrogen site location: inferred from neighbouring sites
*wR*(*F*^2^) = 0.118	H-atom parameters constrained
*S* = 1.01	*w* = 1/[σ^2^(*F*_o_^2^) + (0.0575*P*)^2^ + 0.0575*P*] where *P* = (*F*_o_^2^ + 2*F*_c_^2^)/3
3048 reflections	(Δ/σ)_max_ = 0.001
190 parameters	Δρ_max_ = 0.22 e Å^−^^3^
0 restraints	Δρ_min_ = −0.21 e Å^−^^3^

### Special details

**Table:**

Geometry. All e.s.d.'s (except the e.s.d. in the dihedral angle between two l.s. planes) are estimated using the full covariance matrix. The cell e.s.d.'s are taken into account individually in the estimation of e.s.d.'s in distances, angles and torsion angles; correlations between e.s.d.'s in cell parameters are only used when they are defined by crystal symmetry. An approximate (isotropic) treatment of cell e.s.d.'s is used for estimating e.s.d.'s involving l.s. planes.
Refinement. Refinement of *F*^2^ against ALL reflections. The weighted *R*-factor *wR* and goodness of fit *S* are based on *F*^2^, conventional *R*-factors *R* are based on *F*, with *F* set to zero for negative *F*^2^. The threshold expression of *F*^2^ > σ(*F*^2^) is used only for calculating *R*-factors(gt) *etc*. and is not relevant to the choice of reflections for refinement. *R*-factors based on *F*^2^ are statistically about twice as large as those based on *F*, and *R*- factors based on ALL data will be even larger.

### Fractional atomic coordinates and isotropic or equivalent isotropic displacement parameters (Å^2^)

**Table:**

	*x*	*y*	*z*	*U*_iso_*/*U*_eq_	
C1	0.3269 (3)	0.96011 (19)	0.2888 (2)	0.0449 (5)	
C2	0.4940 (4)	1.0491 (2)	0.3639 (2)	0.0522 (6)	
H2	0.4918	1.0899	0.4510	0.063*	
C3	0.6639 (4)	1.0781 (2)	0.3108 (3)	0.0603 (6)	
H3	0.7754	1.1393	0.3608	0.072*	
C4	0.6651 (4)	1.0152 (2)	0.1836 (3)	0.0579 (6)	
C5	0.5006 (4)	0.9293 (2)	0.1057 (2)	0.0587 (6)	
H5	0.5045	0.8894	0.0186	0.070*	
C6	0.3282 (4)	0.9030 (2)	0.1589 (2)	0.0538 (6)	
H6	0.2125	0.8467	0.1073	0.065*	
C7	0.0960 (3)	0.80999 (19)	0.3412 (2)	0.0434 (5)	
C8	0.2048 (3)	0.58337 (18)	0.25944 (19)	0.0371 (4)	
C9	−0.0095 (3)	0.50477 (19)	0.1946 (2)	0.0414 (5)	
H9A	−0.0449	0.5111	0.1145	0.050*	
H9B	−0.1178	0.5382	0.2481	0.050*	
C10	0.0005 (3)	0.3639 (2)	0.1715 (2)	0.0484 (6)	
H10	−0.1371	0.3139	0.1315	0.058*	
C11	0.0577 (4)	0.3544 (2)	0.2966 (3)	0.0555 (6)	
H11A	0.0617	0.2654	0.2821	0.067*	
H11B	−0.0482	0.3873	0.3521	0.067*	
C12	0.2722 (4)	0.4314 (2)	0.3594 (3)	0.0553 (6)	
H12	0.3091	0.4242	0.4398	0.066*	
C13	0.2620 (3)	0.5721 (2)	0.3845 (2)	0.0479 (5)	
H13A	0.1572	0.6054	0.4407	0.057*	
H13B	0.3972	0.6221	0.4255	0.057*	
C14	0.3707 (3)	0.5295 (2)	0.1712 (2)	0.0467 (5)	
H14A	0.3351	0.5364	0.0914	0.056*	
H14B	0.5073	0.5789	0.2097	0.056*	
C15	0.3806 (3)	0.3894 (2)	0.1469 (2)	0.0524 (6)	
H15	0.4880	0.3562	0.0912	0.063*	
C16	0.1668 (3)	0.3114 (2)	0.0831 (3)	0.0565 (6)	
H16A	0.1725	0.2219	0.0662	0.068*	
H16B	0.1305	0.3176	0.0030	0.068*	
C17	0.4373 (3)	0.3789 (2)	0.2715 (3)	0.0630 (7)	
H17A	0.5744	0.4269	0.3112	0.076*	
H17B	0.4443	0.2899	0.2563	0.076*	
N1	0.1550 (3)	0.92729 (17)	0.34457 (19)	0.0536 (5)	
H1	0.0822	0.9873	0.3838	0.064*	
N2	0.2119 (3)	0.71831 (16)	0.27757 (18)	0.0471 (5)	
H2A	0.3069	0.7428	0.2410	0.057*	
F1	0.8343 (3)	1.04002 (17)	0.12965 (18)	0.0899 (6)	
S1	−0.10933 (9)	0.78832 (5)	0.41551 (6)	0.0557 (2)	

### Atomic displacement parameters (Å^2^)

**Table:**

	*U*^11^	*U*^22^	*U*^33^	*U*^12^	*U*^13^	*U*^23^
C1	0.0514 (11)	0.0311 (10)	0.0520 (14)	0.0071 (8)	0.0101 (10)	0.0159 (9)
C2	0.0629 (13)	0.0420 (12)	0.0466 (13)	0.0011 (10)	0.0050 (11)	0.0140 (10)
C3	0.0571 (13)	0.0512 (14)	0.0647 (17)	−0.0051 (11)	0.0007 (12)	0.0191 (12)
C4	0.0603 (14)	0.0453 (12)	0.0703 (17)	0.0038 (10)	0.0204 (12)	0.0244 (12)
C5	0.0801 (16)	0.0432 (12)	0.0509 (15)	0.0033 (11)	0.0193 (13)	0.0159 (11)
C6	0.0588 (13)	0.0428 (12)	0.0504 (14)	−0.0041 (10)	0.0040 (11)	0.0119 (11)
C7	0.0427 (10)	0.0373 (11)	0.0453 (13)	0.0058 (8)	0.0080 (9)	0.0110 (9)
C8	0.0326 (9)	0.0344 (10)	0.0412 (12)	0.0041 (7)	0.0065 (8)	0.0121 (8)
C9	0.0341 (9)	0.0443 (11)	0.0417 (12)	0.0075 (8)	0.0021 (8)	0.0134 (9)
C10	0.0326 (10)	0.0395 (11)	0.0594 (15)	−0.0005 (8)	0.0024 (9)	0.0076 (10)
C11	0.0512 (12)	0.0471 (12)	0.0744 (17)	0.0048 (10)	0.0152 (12)	0.0302 (12)
C12	0.0545 (13)	0.0573 (14)	0.0604 (15)	0.0069 (10)	−0.0038 (11)	0.0328 (12)
C13	0.0424 (11)	0.0506 (13)	0.0446 (13)	0.0009 (9)	−0.0025 (10)	0.0157 (10)
C14	0.0389 (10)	0.0433 (11)	0.0569 (14)	0.0084 (8)	0.0149 (10)	0.0171 (10)
C15	0.0395 (11)	0.0414 (11)	0.0711 (17)	0.0119 (9)	0.0176 (11)	0.0144 (11)
C16	0.0519 (12)	0.0405 (12)	0.0624 (16)	0.0068 (10)	0.0092 (11)	0.0051 (11)
C17	0.0415 (12)	0.0531 (14)	0.098 (2)	0.0128 (10)	0.0018 (13)	0.0353 (14)
N1	0.0594 (11)	0.0350 (9)	0.0655 (13)	0.0110 (8)	0.0234 (10)	0.0158 (9)
N2	0.0465 (9)	0.0376 (9)	0.0614 (12)	0.0091 (7)	0.0235 (9)	0.0207 (9)
F1	0.0811 (10)	0.0808 (11)	0.1042 (14)	−0.0079 (8)	0.0415 (10)	0.0320 (10)
S1	0.0505 (3)	0.0416 (3)	0.0672 (4)	0.0066 (2)	0.0261 (3)	0.0106 (3)

### Geometric parameters (Å, º)

**Table:**

C1—C2	1.381 (3)	C10—C16	1.533 (3)
C1—C6	1.383 (3)	C10—H10	0.9800
C1—N1	1.421 (3)	C11—C12	1.526 (3)
C2—C3	1.376 (3)	C11—H11A	0.9700
C2—H2	0.9300	C11—H11B	0.9700
C3—C4	1.359 (4)	C12—C17	1.524 (3)
C3—H3	0.9300	C12—C13	1.530 (3)
C4—F1	1.362 (3)	C12—H12	0.9800
C4—C5	1.364 (4)	C13—H13A	0.9700
C5—C6	1.380 (3)	C13—H13B	0.9700
C5—H5	0.9300	C14—C15	1.526 (3)
C6—H6	0.9300	C14—H14A	0.9700
C7—N2	1.345 (2)	C14—H14B	0.9700
C7—N1	1.351 (3)	C15—C17	1.514 (4)
C7—S1	1.687 (2)	C15—C16	1.527 (3)
C8—N2	1.473 (2)	C15—H15	0.9800
C8—C13	1.524 (3)	C16—H16A	0.9700
C8—C14	1.533 (3)	C16—H16B	0.9700
C8—C9	1.535 (3)	C17—H17A	0.9700
C9—C10	1.537 (3)	C17—H17B	0.9700
C9—H9A	0.9700	N1—H1	0.8600
C9—H9B	0.9700	N2—H2A	0.8600
C10—C11	1.514 (4)		
			
C2—C1—C6	119.4 (2)	H11A—C11—H11B	108.2
C2—C1—N1	120.3 (2)	C17—C12—C11	109.3 (2)
C6—C1—N1	120.3 (2)	C17—C12—C13	109.9 (2)
C3—C2—C1	120.5 (2)	C11—C12—C13	109.09 (18)
C3—C2—H2	119.7	C17—C12—H12	109.5
C1—C2—H2	119.7	C11—C12—H12	109.5
C4—C3—C2	118.5 (2)	C13—C12—H12	109.5
C4—C3—H3	120.7	C8—C13—C12	109.58 (18)
C2—C3—H3	120.7	C8—C13—H13A	109.8
C3—C4—F1	119.4 (2)	C12—C13—H13A	109.8
C3—C4—C5	122.7 (2)	C8—C13—H13B	109.8
F1—C4—C5	117.8 (2)	C12—C13—H13B	109.8
C4—C5—C6	118.5 (2)	H13A—C13—H13B	108.2
C4—C5—H5	120.7	C15—C14—C8	110.05 (17)
C6—C5—H5	120.7	C15—C14—H14A	109.7
C5—C6—C1	120.2 (2)	C8—C14—H14A	109.7
C5—C6—H6	119.9	C15—C14—H14B	109.7
C1—C6—H6	119.9	C8—C14—H14B	109.7
N2—C7—N1	115.40 (17)	H14A—C14—H14B	108.2
N2—C7—S1	125.04 (16)	C17—C15—C14	109.59 (19)
N1—C7—S1	119.56 (14)	C17—C15—C16	109.8 (2)
N2—C8—C13	111.51 (17)	C14—C15—C16	109.42 (18)
N2—C8—C14	105.19 (16)	C17—C15—H15	109.3
C13—C8—C14	109.26 (17)	C14—C15—H15	109.3
N2—C8—C9	112.54 (16)	C16—C15—H15	109.3
C13—C8—C9	109.88 (16)	C15—C16—C10	108.83 (18)
C14—C8—C9	108.27 (16)	C15—C16—H16A	109.9
C8—C9—C10	109.31 (16)	C10—C16—H16A	109.9
C8—C9—H9A	109.8	C15—C16—H16B	109.9
C10—C9—H9A	109.8	C10—C16—H16B	109.9
C8—C9—H9B	109.8	H16A—C16—H16B	108.3
C10—C9—H9B	109.8	C15—C17—C12	109.70 (18)
H9A—C9—H9B	108.3	C15—C17—H17A	109.7
C11—C10—C16	109.86 (19)	C12—C17—H17A	109.7
C11—C10—C9	109.71 (18)	C15—C17—H17B	109.7
C16—C10—C9	109.11 (19)	C12—C17—H17B	109.7
C11—C10—H10	109.4	H17A—C17—H17B	108.2
C16—C10—H10	109.4	C7—N1—C1	125.69 (17)
C9—C10—H10	109.4	C7—N1—H1	117.2
C10—C11—C12	109.87 (19)	C1—N1—H1	117.2
C10—C11—H11A	109.7	C7—N2—C8	131.37 (17)
C12—C11—H11A	109.7	C7—N2—H2A	114.3
C10—C11—H11B	109.7	C8—N2—H2A	114.3
C12—C11—H11B	109.7		
			
C6—C1—C2—C3	−2.1 (3)	C11—C12—C13—C8	−60.2 (2)
N1—C1—C2—C3	177.9 (2)	N2—C8—C14—C15	179.09 (18)
C1—C2—C3—C4	−1.4 (4)	C13—C8—C14—C15	59.3 (2)
C2—C3—C4—F1	−178.3 (2)	C9—C8—C14—C15	−60.4 (2)
C2—C3—C4—C5	3.3 (4)	C8—C14—C15—C17	−59.6 (2)
C3—C4—C5—C6	−1.6 (4)	C8—C14—C15—C16	60.8 (3)
F1—C4—C5—C6	179.9 (2)	C17—C15—C16—C10	59.9 (2)
C4—C5—C6—C1	−1.9 (4)	C14—C15—C16—C10	−60.4 (3)
C2—C1—C6—C5	3.7 (3)	C11—C10—C16—C15	−59.5 (3)
N1—C1—C6—C5	−176.2 (2)	C9—C10—C16—C15	60.9 (3)
N2—C8—C9—C10	176.37 (17)	C14—C15—C17—C12	59.6 (2)
C13—C8—C9—C10	−58.7 (2)	C16—C15—C17—C12	−60.6 (2)
C14—C8—C9—C10	60.5 (2)	C11—C12—C17—C15	59.8 (3)
C8—C9—C10—C11	59.0 (2)	C13—C12—C17—C15	−59.8 (3)
C8—C9—C10—C16	−61.4 (2)	N2—C7—N1—C1	−0.8 (3)
C16—C10—C11—C12	59.6 (2)	S1—C7—N1—C1	178.58 (18)
C9—C10—C11—C12	−60.3 (2)	C2—C1—N1—C7	−117.2 (3)
C10—C11—C12—C17	−59.4 (2)	C6—C1—N1—C7	62.7 (3)
C10—C11—C12—C13	60.7 (2)	N1—C7—N2—C8	176.0 (2)
N2—C8—C13—C12	−174.86 (16)	S1—C7—N2—C8	−3.4 (4)
C14—C8—C13—C12	−59.0 (2)	C13—C8—N2—C7	−64.7 (3)
C9—C8—C13—C12	59.6 (2)	C14—C8—N2—C7	177.0 (2)
C17—C12—C13—C8	59.6 (2)	C9—C8—N2—C7	59.3 (3)

### Hydrogen-bond geometry (Å, º)

**Table:**

*D*—H···*A*	*D*—H	H···*A*	*D*···*A*	*D*—H···*A*
N1—H1···S1^i^	0.86	2.67	3.3939 (19)	142

Symmetry code: (i) −*x*, −*y*+2, −*z*+1.
